# Respiratory disturbances and high risk of sudden death in the neonatal connexin‐36 knockout mouse

**DOI:** 10.14814/phy2.15109

**Published:** 2021-11-10

**Authors:** Leonel F. Pérez‐Atencio, Ana M. Casarrubios, José M. Ibarz, Juan A. Barios, Cristina Medrano, David Pestaña, David L. Paul, Luis C. Barrio

**Affiliations:** ^1^ Unit of Experimental Neurology “Ramón y Cajal” Hospital (IRYCIS) Madrid Spain; ^2^ Biomedical Neuroengineering Research Group (nBio) Systems Engineering and Automation Department of Miguel Hernández University Elche Spain; ^3^ Anesthesiology Service “Ramón y Cajal” Hospital (IRYCIS) Madrid Spain; ^4^ Department of Neurobiology Harvard Medical School Boston Massachusetts USA; ^5^ Centro de Tecnología Biomédica de la Universidad Politécnica Madrid Spain

**Keywords:** cardiorespiratory coupling, connexin36, electrical synapses, respiratory center, SIDS

## Abstract

Neural circuits at the brainstem involved in the central generation of the motor patterns of respiration and cardiorespiratory chemoreflexes organize as cell assemblies connected by chemical and electrical synapses. However, the role played by the electrical connectivity mainly mediated by connexin36 (Cx36), which expression reaches peak value during the postnatal period, is still unknown. To address this issue, we analyzed here the respiratory phenotype of a mouse strain devoid constitutively of Cx36 at P14. Male Cx36‐knockout mice at rest showed respiratory instability of variable degree, including a periodic Cheyne–Stokes breathing. Moreover, mice lacking Cx36 exhibited exacerbated chemoreflexes to normoxic and hypoxic hypercapnia characterized by a stronger inspiratory/expiratory coupling due to an increased sensitivity to CO_2_. Deletion of Cx36 also impaired the generation of the recurrent episodes of transient bradycardia (ETBs) evoked during hypercapnic chemoreflexes; these EBTs constituted a powerful mechanism of cardiorespiratory coupling capable of improving alveolar gaseous exchange under hypoxic hypercapnia conditions. Approximately half of the homo‐ and heterozygous Cx36KO, but none WT, mice succumbed by respiratory arrest when submitted to hypoxia‐hypercapnia, the principal exogenous stressor causing sudden infant death syndrome (SIDS). The early suppression of EBTs, which worsened arterial O_2_ saturation, and the generation of a paroxysmal generalized clonic‐tonic activity, which provoked the transition from eupneic to gasping respiration, were the critical events causing sudden death in the Cx36KO mice. These results indicate that Cx36 expression plays a pivotal role in respiratory control, cardiorespiratory coordination, and protection against SIDS at the postnatal period.

## Graphical abstract

Neonatal Cx36‐knockout mice show breathing instability at rest, exacerbated chemoreflexes, disturbances in the mechanisms of cardiorespiratory coupling, and an elevated risk of sudden infant death. The principal determinants leading to the sudden infant death in the Cx36‐knockout mice submitted to hypoxic‐hypercapnic stress were the oxygen desaturation caused by the early suppression of mechanisms of cardiorespiratory coordination and the generation of a paroxysmal generalized clonic‐tonic activity that changes of respiratory pattern from eupneic to a gasping mode.

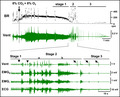

## 1 INTRODUCTION

Breathing is a primal neural process that through the adjustment of the rate and depth of ventilation maintains the O_2_ and CO_2_ levels at physiological values. Neural circuits of the brainstem responsible for generating the central motor pattern of respiration and chemoreflex responses are organized as serially arrayed networks connected by chemical but also by electrical synapses (Dean et al., 2002; Parenti et al., 2000; Solomon, 2003). Electrical synapses constitute key elements in synaptic circuitry, governing the collective activity of electrically coupled networks. Currently, gap junction protein connexin‐36 (Cx36) is considered the principal component of electrical synapses in multiple brain regions with a preferential expression in inhibitory interneurons (Belluardo et al., 2000; Deans et al., 2001; Nagy et al., 2018). The generation of Cx36 null mice confirmed the idea that electrical connectivity promotes the synchronous activity of neuronal networks and clustering of coherent rhythmic activities (Deans et al., 2001; Hormuzdi et al., 2001; Kraft et al., 2020), and revealed functional deficits in multiple neuronal networks, including visual, motor, cortex synchronization, spindle rhythm, circadian rhythm, oxytocin release, gamma rhythm, and memory impairments among others (Nagy et al., 2018). At brainstem, a moderate to high labeling of Cx36 mRNA and protein was visualized in nuclei involved in the generation of respiratory rhythm, as preBötzinger and Bötzinger complexes (preBotC and BotC), in central chemoreception as the retrotrapezoid nucleus (RTN), the nucleus of solitary tract, dorsal nucleus of raphe and locus coeruleus, and in the cardiorespiratory coordination as the ambiguous nucleus and dorsal motor nucleus of vagus (Parenti et al., 2000; Solomon, 2003). Electrical connectivity in some of these nuclei has been also confirmed by dye and electrical coupling, and in some instances, this coupling has been reported to be pH insensitive to hypercapnic acid (Dean et al., 2002), which is consistent with the uncoupling of Cx36 channels upon alkalinization (González‐Nieto et al., 2008). Like in other brain areas, Cx36 expression at brainstem nuclei are developmentally regulated with a peak expression during the first weeks of postnatal life (Belluardo et al., 2000; Solomon, 2003). However, the functional contribution of electrical connectivity mediated by Cx36 in the respiratory control and autonomic regulation of cardiorespiratory coupling in this critical period of life remains to be determined. To address this issue, we studied the respiratory phenotype of a mouse strain devoid constitutively of Cx36 (Deans et al., 2001). Because an important subset of sudden infant death syndromes (SIDS) is due to central defects in the breathing control and mechanisms of cardiorespiratory coordination (Harper & Kinney, 2010), the risk of sudden death in the Cx36‐knockout mouse was also evaluated.

## 2 MATERIALS AND METHODS

### 2.1 Animals

Male wild‐type and homozygous (Cx36^–^/^–^) and heterozygous (Cx36^+^/^–^) connexin36 knockout mice (WT and Cx36KO) from postnatal day 14 (P14) were used in this study; the deletion of the Cx36 gene was previously described (Deans et al., 2001). All animals were housed in cages at 21–23°C under a 12:12 h light‐dark cycle and provided food and water ad libitum. Animal care, use, and experimental protocols were approved by the Local Ethics Committee (PROEX 165/16), responsible for the correct application of the order 86/609/CEE (Spanish order 1201/2005). The procedure for euthanasia of animals was anesthetic overdose of isoflurane with 100% O_2_ followed by dislocation of the neck.

### 2.2 Experimental design and electrophysiological recordings

Conscious mice were placed in a face‐down position into a gaseous exchange chamber (6 L) with constant airflow (6 L/min); mice were partially immobilized by their extremities with the adhesive tape to avoid escape reactions and preserve the stability of the electrophysiological recordings. During the experimental procedures, EMG activity of inspiratory and expiratory musculatures, thoracic respiratory excursions, ECG activity, arterial saturation of O_2_, partial pressure of CO_2_, and pulse distention were continuously monitored. Bipolar platinum–iridium hook electrodes (1–1.5 MΩ) insulated except for active tip were implanted under anesthesia with 2% isoflurane in 100% O_2_ at the abdominal surface of the diaphragm and 9th interosseous space for recording the electromyographic activity of the diaphragm (EMG_D_) and inspiratory external and expiratory internal intercostal muscles (EMG_I_); recovery from anesthesia took place 30–60 min when breathing frequency returned to basal values. The respiratory chest wall motion, proportional to the volume of air inhaled and exhaled detected by a thermistor fluxmeter, was measured by using a home‐made device based on Hall’s effect with a magnet and a magnetic sensor on each side of the thorax. Pulse oximetry with a neck collar was used to measure arterial saturation of O_2_ (SpO_2_) and oxyhemoglobin (OHb) pulse‐to‐pulse raw infrared light AC‐coupled signal (MouseOx, Starr Life Science); this device also provided pulse distention of carotid arteries. The partial pressure of CO_2_ (PtcCO_2_) was measured with a noninvasive transcutaneous sensor placed on the abdomen skin (V‐sign Sensor 2, SenTec). SpO_2_ and PtcCO_2_ calibrations was obtained from a blood sample of the tail. Finally, ECG activity was recorded with a monopolar surface electrode placed on the parasternal intercostal space. After 30 min habituation breathing medicinal air, animals were exposed to a series of near square‐wake gas challenges of hypercapnia in normoxia (4%, 8%, or 12% CO_2_ with 21% O_2_ and supplemented with N_2_), severe hypoxia in normocapnia (8% O_2_ with 92% N_2_) and asphyxia, by combining hypoxia and hypercapnia (8% O_2_ with 4%, 8% and 12% CO_2_ and supplemented with N_2_); each stimulus was of 10 min duration and followed by periods of at least 30 min with medicinal air for full recovery basal cardiorespiratory status.

### 2.3 Data analysis

Amplified AC signals of chest wall motion, ECG, and EMG (×10, ×100, and ×1000) were filtered (0.1–50 Hz and 15–5000 Hz) were sampled at 2 kHz (Biomedical Workbench), and off‐line processing with Spike2, R, and MATLAB software. Raw diaphragmatic and intercostal EMG activities were full‐wave rectified and integrated with a time constant of 5 ms (∫EMG_D_ and ∫EMG_I_). Peak‐intensity of inspiratory and expiratory motor activities was calculated from 20 breathing cycles and normalized relative to their values at rest (∆∫EMG_D_ and ∆∫EMG_I_). The period of breathing cycle (BP) was divided into an inspiratory phase (I), defined by the duration of the diaphragm or external intercostal activities, and an expiratory phase (*E*) with three components: an active expiration (*E*_2_), corresponding to activation of internal intercostal muscles, and two intervals of passive expiration, a post‐inspiratory and another pre‐inspiratory silence preceding and following *E*_2_, termed *E*_1_ and *E*_3_. From recordings of respiratory chest wall motion (Vent) were obtained breathing rate (BR) and valley‐peak ventilatory amplitude (VA); the normalized VA concerning basal value was used as an index of tidal volume (∆VT) between rest and stimulus‐response. To visualize the dynamics of recurrent episodes of transient bradycardia (ETBs), ECG signal‐based Poincaré plots for successive beating intervals (RR*_n_* and RR*_n_*_+1_) were constructed after using an algorithm for detecting R‐waves of QRS complex. A point process with ETB onsets was generated from the beginning of longest RR interval for averaging heart rate change during ETBs and to determine the phase of ETBs occurrence within the breathing cycle; for that purpose, the period of cycles was normalized using Hilbert’s transform which converted the periods of variable duration in phase degrees.

### 2.4 Statistical analyses

Data were represented as the mean and standard deviation (SD). Statistical comparisons were assessed with Student’s *t*‐test or Mann–Whitney correction and ANOVA or Bonferroni test, depending on the fulfilment of the hypothesis of normality of data; the log‐rank test used to compare Kaplan–Meier curves; the actual value of statistical significance (*p*) was stated for each comparison. This statistical analysis was performed using Sigma Plot (Hearne Scientific) and MATLABv.2008 (Mathworks) software.

## 3 RESULTS

### 3.1 Respiratory disturbances in the neonatal Cx36‐KO mice

**FIGURE 1:**
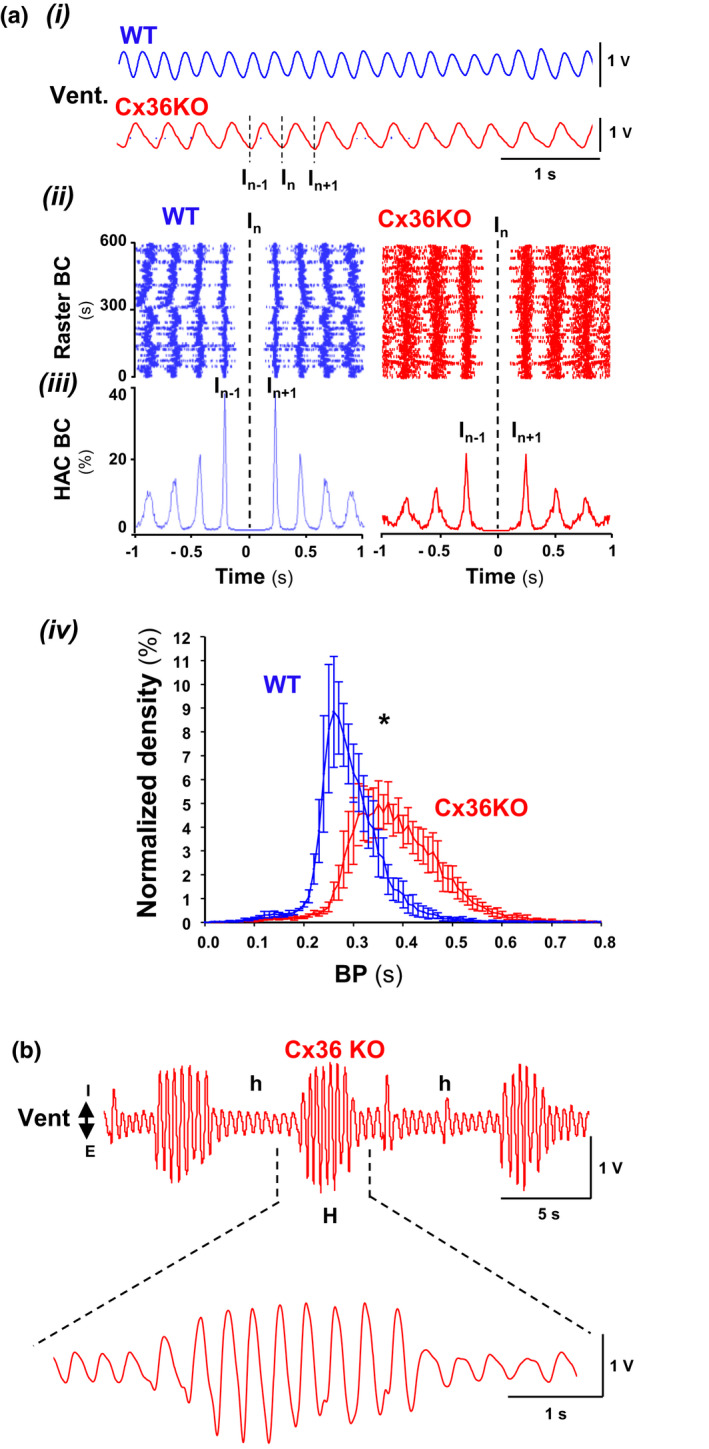
Breathing instability in the neonatal Cx36KO mice at rest. (a) Breathing of most Cx36KO mice at P14 (86%) exhibited a slower and more irregular eupneic respiration at rest; (i‐iii) example of raw chest motion records with the corresponding raster and histogram of autocorrelation of breathing cycles, BC; (iv) average of the normalized histogram of breathing periods, BP (*N* = 10 per group, 10 min; *p < 0.0001, ANOVA). (b) The remaining Cx36KO mice (14%) showed a periodic Cheyne–Stokes breathing with recurrent episodes of hypopneas (*h*) and crescendo‐decrescendo hyperpneas (*H*)

Neonatal (P14) Cx36‐knockout male mice (Cx36KO) breathing room air showed respiratory instability of variable degree; most of them (28 out 33; 86%) exhibited a slower respiratory frequency (BR) and more irregular rhythm (BR = 189.11 ± 5.74 vs. 213.41 ± 6.86 rmp and CV = 0.24 ± 0.01 vs. 0.18 ± 0.01% for Cx36KO and WT; *N* = 30 for each group, *p* = 0.0086 and *p* = 0.0006, *t*‐Student test; Figure 1ai–iv). The remaining Cx36KO mice (5 out 33; 14%) presented a persistent periodic respiration type Cheyne–Stokes with recurrent episodes of hypopneas and crescendo‐decrescendo hyperpneas with an oscillatory period of 0.18 ± 0.04 Hz (Figure 1b); this periodic respiration never observed in WT mice. Cx36KO mice with Cheyne–Stokes respiration were discarded in subsequent studies.

**FIGURE 2:**
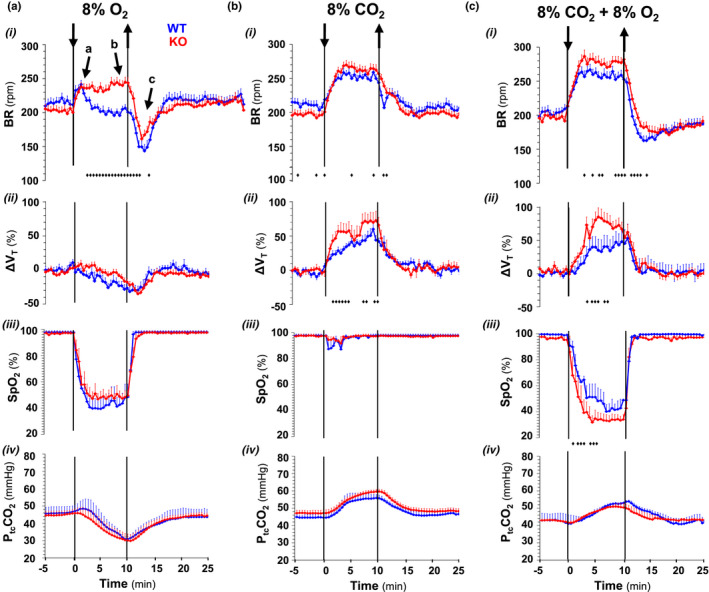
Exacerbated chemoreflexes to hypoxia (a), normoxic and hypoxic hypercapnia (b and c) in the neonatal Cx36‐knockout mice. Superimposed responses of wild type and Cx36‐KO mice (in blue and red): (i) breathing rate (BR); (ii) index of tidal volume (∆VT), (iii) arterial O_2_ saturation (SpO_2_), and (iv) transcutaneous CO_2_ partial pressure (PtcCO_2_). Cx36‐KO mice responded to hypoxia with a sustained increase of BR instant of a fully adapted response of WT mice, and to hypercapnia in normoxia and hypoxia with greater increases in BR and ∆VT. Note that while hypoxia (8% O_2_) induced similar O_2_ desaturation in both genotypes, hypoxic hypercapnia (8% O_2_ and 8% CO_2_) provoked a more pronounced O_2_ desaturation in Cx36KO than WT mice. PtcCO_2_ decreased during hypoxia and increased during hypercapnia in normoxia and hypoxia equally in the two groups (*N* = 24 per group; in A‐i ♦, *p* = 0.0215‐<0.0001; in B‐i ♦, *p* = 0.0495–0.0045; in C‐i ♦, *p* = 0.0457–0.0098; in B‐ii ♦, *p* = 0.0448–0.0078; in C‐ii ♦, *p* = 0.0484–0.0140, and in C‐iii ♦, *p* = 0.0492–0.0015; *t*‐Student test)

**FIGURE 3:**
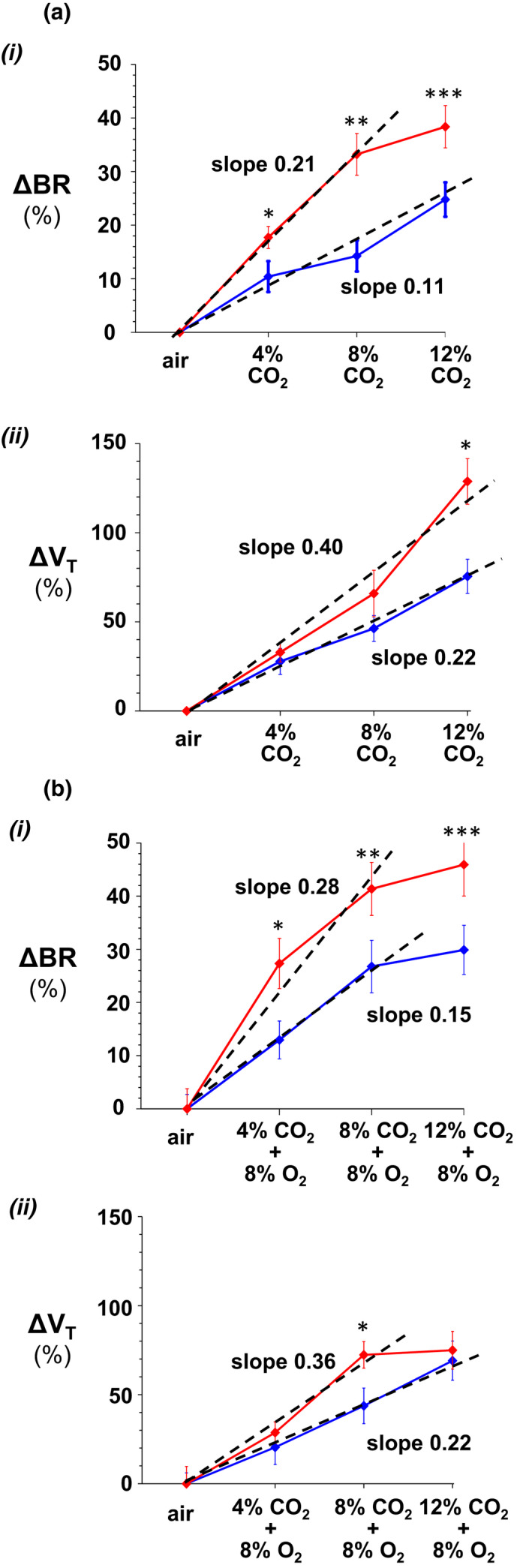
Increased CO_2_ sensitivity of neonatal Cx36KO. Wild type and Cx36KO mice (in blue and red) were submitted to increasing concentrations of CO_2_ in inhaled air (4%, 8%, and 12%) in normoxia (a) and 8% O_2_ hypoxia (b); (i and ii) breathing rate (∆BR) and index of tidal volume (∆*V*_T_; last 2 of 10 min stimulus) normalized relative to basal values. The slopes of normalized breathing rate (∆HR) and index of tidal volume (∆*V*_T_) as function of CO_2_ concentration were ≈twofold greater in Cx36KO than WT mice for both normoxic and hypoxic hypercapnia (*N* = 15 per group; in A‐i *p = 0.0478; **p = 0.0005 and ***p = 0.0125; in A‐ii *p = 0.0024; in B‐i *p = 0.0216; **p = 0.0216; ***p = 0.0417; in B‐ii, *p = 0.0291; *t*‐Student test)

Next, chemoreflexes to hyperoxia and hypoxia, and hypercapnia in normoxia and hypoxia were analyzed. Hyperoxia (100% inspired O_2_) caused a similar small respiratory depression in both genotypes (BR = 181.62 ± 36.37 vs. 181.97 ± 28.65 rmp for Cx36KO and WT; *N* = 5 per group, *p* = 0.9871, *t*‐Student test). However, Cx36KO mice at P14 responded to severe hypoxia (from 21% to 8% O_2_ in inhaled air) with a sustained increment of respiratory frequency instead of fully adapted response of WT mice and other species at this age (Bissonnette, 2000), and less respiratory depression post‐hypoxia (Figure 2ai, arrows a–c). Hypoxia (8% O_2_) induced in both mice groups a similar rapid drop in SaO_2_ to steady state values of 42.45 ± 8.46% and 49.73 ± 5.76% in WT and Cx36KO (*N* = 24, *p* = 0.4804, *t*‐Student test) and, secondarily (Basting et al., 2015), a similar reduction of PtcCO_2_ (from 45.04 ± 2.53 to 30.90 ± 4.07 mmHg in WT and from 45.96 ± 3.70 to 31.88 ± 1.75 mmHg for Cx36KO; *N* = 25, *p* = 0.8258, *t*‐Student test; Figure 2a iii and iv). However, this reduction in CO_2_ was less efficient to adapt and depress breathing in Cx36KO than WT mice, suggesting that the sensitivity to CO_2_ may be increased in mice lacking Cx36. To this regard, Cx36‐KO mice responded to increasing concentrations of CO_2_ in inhaled air (to 4%, 8%, and 12%) alone and in combination with hypoxia (8% O_2_) with greater increases of respiratory frequency and tidal volume index than WT mice (Figure 2b,ci and ii). The CO_2_ sensitivity, defined by the slope of normalized increments of the breathing rate (∆HR) and index of tidal volume (∆VT) as a function of CO_2_ concentration, was ≈twofold greater in Cx36KO than WT mice for the stimuli of hypercapnia in normoxia or hypoxia (Figure 3a,b). However, this greater ventilatory effort of Cx36KO mice in hypoxic hypercapnia was paradoxically associated with a significantly greater drop in SaO_2_ up to 42.63 ± 1.14% versus 52 ± 1.89% of WT (*N* = 25, *p* = 0.0001, *t*‐Student test; Figure 2Ciii).

**FIGURE 4:**
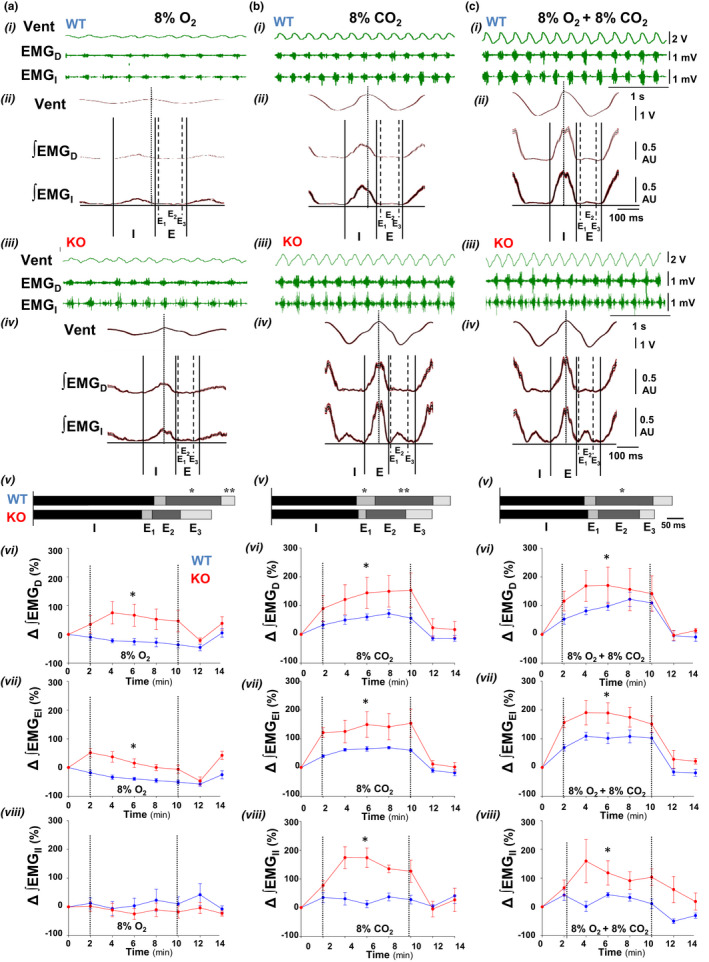
Respiratory motor pattern of Cx36KO versus WT mice during hypoxia (a), and normoxic and hypoxic hypercapnia (b and c). (i and iii) Representative raw records of respiratory chest motion (Vent), diaphragm, and intercostals activities (EMG_D_ and EMG_I_), (ii and iv) averages (20 respiratory cycles) of Vent and rectified EMGs (∫EMG_D_ and ∫EMG_I_) from WT and Cx36KO mice (in blue and red), (v) averaged duration of inspiratory phase (I) and expiratory phase (*E*), subdivided in one active (*E*_2_) and two passive post‐inspiratory and pre‐inspiratory expiratory components (*E*_1_ and *E*_3_), (vi–viii) peak‐intensity of rectified inspiratory activity of the diaphragm (∆∫EMG_D_) and external intercostal muscles (∆∫EMG_EI_), and expiratory activity of internal intercostals (∆∫EMG_II_) normalized relative to their values at rest. Cx36KO mice responded to hypoxia with a greater inspiratory activity and shorter breathing cycles and expiratory phases, and to normoxic and hypoxic hypercapnia with more intensive and phasic inspiratory and expiratory motor activities in anti‐phase (*N* = 15 per group in A–C v; in A‐v *p < 0.0001, and ***p = 0.0082; in B‐v *p = 0.0002, and **p = 0.0008; and in C‐v *p = 0.0001 *t*‐Student test. *N* = 6 per group in A–C vi‐viii; in A‐vi, *, *p* < 0.0001 and in A‐vii, *p < 0.0001; in B‐vi, *p = 0.0008; in B‐vii, *p < 0.0001, and in B‐viii, *p < 0.0001; in C‐vi, *p = 0.0316; in C‐vii, *p < 0.0001 and in C‐viii, *p < 0.0001, ANOVA)

The analysis of the EMG activity of inspiratory and expiratory musculature allowed to identify the reconfigurations of the respiratory motor pattern underlying the exacerbated chemoreflexes observed in the Cx36KO mice. In comparison, Cx36KO mice responded to hypoxia with greater and sustained inspiratory activity of the diaphragm and external intercostals, and a larger shortening of the expiratory phase (Figure 4a,i–viii). Expiratory musculature did not contribute significatively to the steady‐state response to hypoxia but it played a pivotal role in the hypercapnic chemoreflexes; in comparison, the Cx36KO mice responded to hypercapnia‐normoxia and hypercapnia‐hypoxia with a more intensive phasic activation of expiratory and inspiratory musculatures and a briefer active expiratory E2 component (Figure 4b,ci–viii).

### 3.2 Disturbances in the mechanisms of cardiorespiratory coupling

**FIGURE 5:**
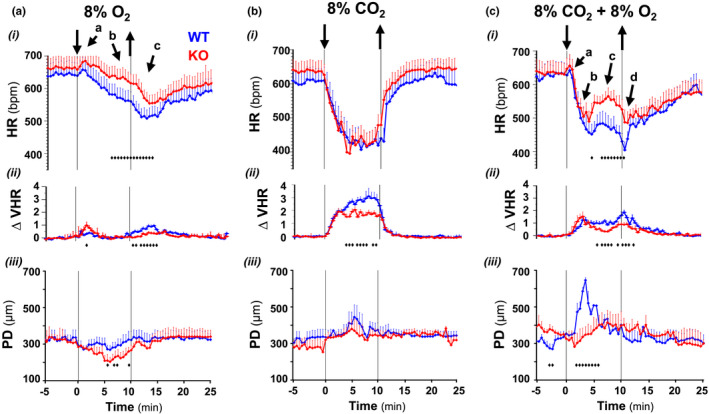
Cardiovascular disturbances during chemoreflexes to hypoxia (a), and normoxic and hyperoxic hypercapnia (b and c) in the neonatal Cx36‐knockout mice. Superimposed responses of wild type and Cx36‐KO mice (in blue and red): (i) heart rate (HR), (ii) heart rate variability normalized relative basal value (∆VHR), and (iii) pulse distention (PD). Cx36KO mice responded to hypoxia with a similar initial tachycardia followed by a smaller slow building‐up bradycardia and early trough post‐hypoxia (arrows a‐c); hypercapnia in normoxia provoked similar sharp bradycardia in WT and Cx36KO mice but cardiac variability was less pronounced in Cx36KO mice; during hypoxic hypercapnia, the initial small tachycardia was similar in both genotypes but the sharp bradycardia was damped and the subsequent partial recovery of HR increased more in Cx36KO mice (arrows a‐c); PD decreased more during hypoxia and increased less during hypoxic hypercapnia in Cx36KO than WT mice (*N* = 25 per group; in A‐i ♦, *p* = 0.0471–0.0012; in A‐ii ♦, *p* = 0.02280–0.0004; in A‐iii ♦, *p* = 0.0485–0.0422; in B‐ii ♦, *p* = 0.0389–0.0005; in C‐i ♦, *p* = 0.0491–0.0168; in C‐ii ♦, *p* = 0.0369‐ < 0.0001, and in C‐iii ♦, *p* = 0.0497‐ < 0.0001; *t*‐Student test)

Chemoreflexes involve important adjustments of cardiovascular function in coordination with respiratory responses by regulating central cardiovagal and sympathetic outflows (Guyenet, 2014). In this regard, Cx36 deletion also altered the cardiovascular response during hypoxic and hypercapnic reflexes. Hypoxia induced a biphasic sympathetic‐parasympathetic effect on heart rate causing a small initial acceleration followed by a progressive bradycardia that increased transiently during reoxygenation before slowly returning to basal value (Figure 5ai arrows a–c); in comparison, the heart rate of Cx36‐KO mice decreased less during the steady‐state response to hypoxia and reoxygenation, reflecting a reduction in the parasympathetically‐mediated response that, at the vascular level, resulted in a smaller distension of pulse (Figure 5aiii). Hypercapnia in normoxia induced on average a similar rapid and potent bradycardic effect in both genotypes (Figure 5bi and ii). Hypercapnia‐hypoxia caused a more complex regulation of heart rate since the initial tachycardia of hypoxia combined with the marked bradycardia induced by hypercapnia followed by a partial later recovery of the heart rate before fell again during reoxygenation as in hypoxia (Figure 5ci arrows a–d); in comparison, Cx36KO mice responded with a smaller bradycardia and a more pronounced late heart rate recovery, and less heart rate drop during reoxygenation. Concomitant with the abrupt initial drop of heart rate and its subsequent partial recovery, the pulse distention in the WT mice first increased suddenly and then declined (Figure 5ciii); this vascular response was mostly absent in the case of Cx36KO mice. All these differential effects in heart rate and pulse distension indicate that the parasympathetic response in mice lacking Cx36 was also impaired during hypoxic hypercapnia reflex.

**FIGURE 6:**
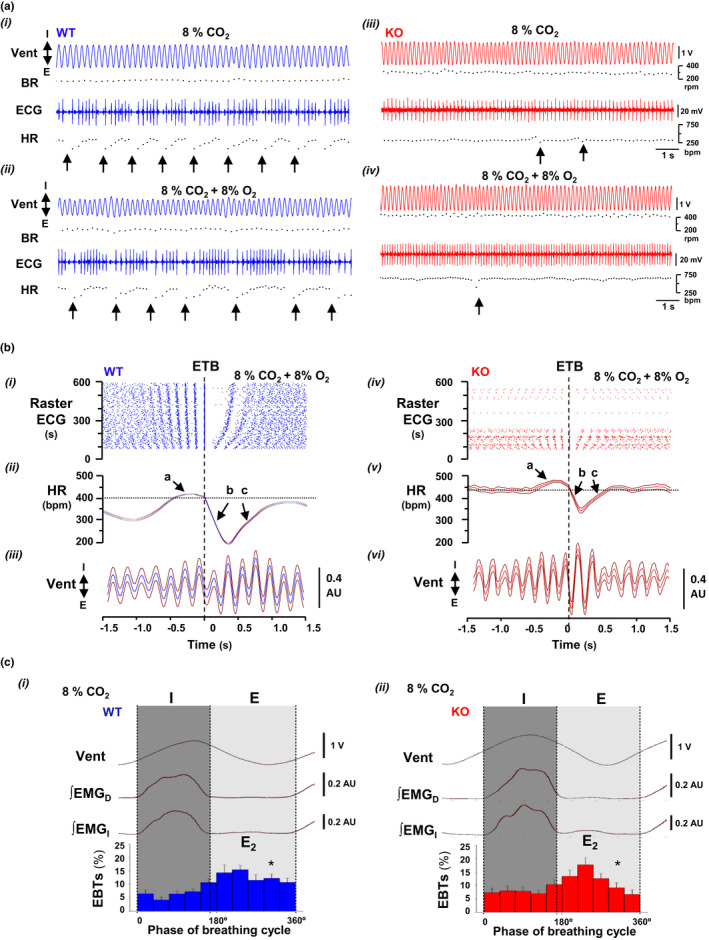
Cardiorespiratory coupling during normoxic and hypoxic hypercapnia. (A) Raw records from WT and Cx36KO mice (in blue and red) of respiratory motion (Vent), cardiac activity (ECG), and instantaneous breathing and heart rates (BR and HR) selected from the last 2 of 10 min exposition to hypercapnia‐normoxia (i and iii) and hypercapnia‐hypoxia (ii and iv) showing repetitive episodes of transient bradycardia (ETBs; arrows). (B) Raster of ETBs generated by WT and Cx36KO mice (right and left) along 10 min of hypoxic hypercapnia (i and iv), heart rate average of ETBs generated during last 2 min (HR, ii and v), cross‐correlation of respiration (Vent) with the onset of ETBs (iii and vi). HR during ETBs first increased slightly, and then dropped abruptly and recovered slowly (arrows a–c); in comparison, Cx36KO mice generated ETBs of smaller intensity and its number declined rapidly with the time of exposition to hypoxic hypercapnia. ETBs onset was phase‐locked with the expiration phase of the breathing cycle in both genotypes. (C) Probability of ETBs occurrence within the normalized breathing cycles of WT and Cx36KO mice (i and ii); Vent, ventilatory motion, and ∫EMG_D_ and ∫EMG_I_, rectified activities of the diaphragm and intercostal muscles. Note that the maximal probability of ETB generation occurred during active expiration (*E*_2_) (*N* = 8 per group; in C‐i *p < 0.0001, and in C‐ii *p = 0.0011; ANOVA)

**FIGURE 7:**
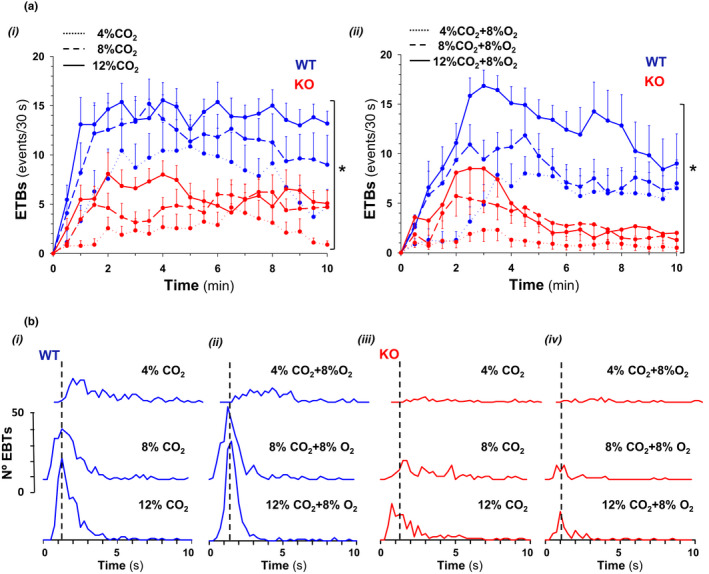
Effect of CO_2_ concentration in the generation of ETBs. (A) Number of ETBs generated by WT and Cx36KO mice (blue and red) during 10 min of hypercapnia alone and in combination with hypoxia (i and ii). During hypercapnia‐normoxia, ETBs number increased rapidly after stimulus starting to greater steady‐state values as larger CO_2_ concentrations were applied in inhaled air; in comparison, Cx36KO mice generated significantly fewer ETBs. During hypercapnia‐hypoxia, the EBTs rate tended to decline with the time of exposition to hypoxic hypercapnia but more markedly in the case of Cx36 (*N* = 12 per group; in A i and ii *p < 0.0001; ANOVA). (B) Example of interval histograms of ETBs generated by WT and Cx36KO mice (blue and red) in hypercapnia‐normoxia (i and ii) and hypercapnia‐hypoxia (iii and iv). Note that ETBs tended to generate at a fixed interval (broken line) which probability increased with CO_2_ level

**FIGURE 8:**
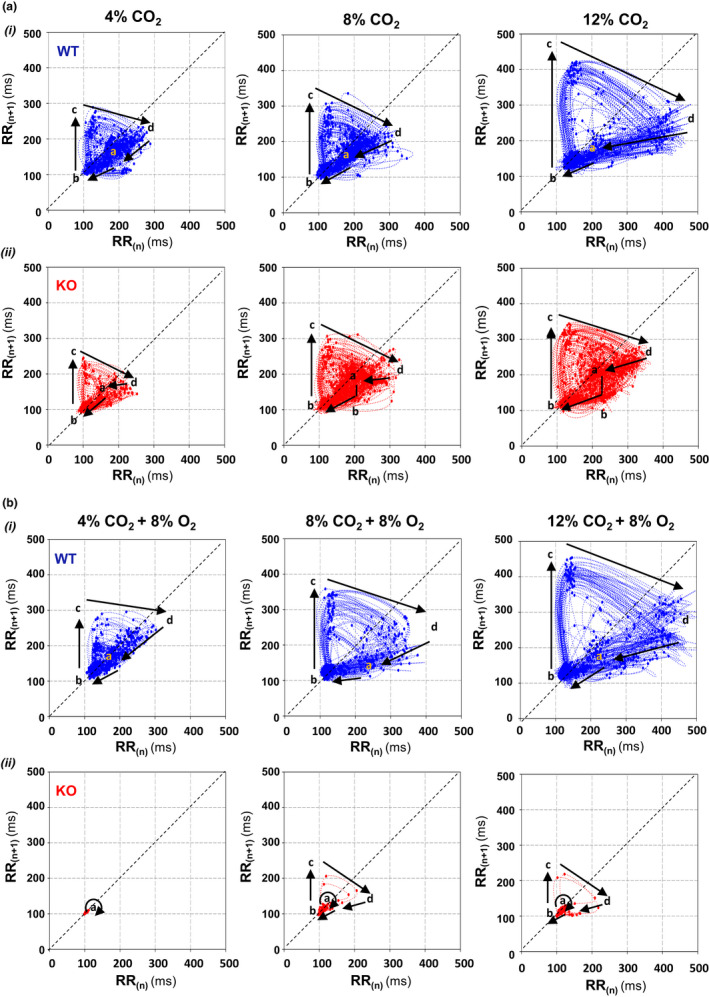
ECG signal‐based Poincaré plots of ETBs as a function of CO_2_ concentration. Poincaré diagrams (RR_n_ vs. RR*_n_*_+1_ in *x *and *y*‐axis) constructed with steady‐state data of ETBs (last 2 min) in normoxic and hypoxic hypercapnia (A and B) from WT and Cx36KO mice (i and ii). Dynamics of interval sequence during ETBs showed an initial small shortening of RR interval (*a*→*b*) followed by a longer lengthening of next RR (*b*→*c*), and then a progressive recovery toward initial RR value in successive cardiac cycles (*c*→*d*→*a*); this elongation of RR increased similarly with CO_2_ concentration up to double and triple of the basal RR interval for WT and Cx36KO mice in normoxic hypercapnia and WT mice in hypoxic hypercapnia; note that in the case of the Cx36KO mice this bradycardic effect was almost completely absent during hypoxic hypercapnia

Hypercapnia in normoxia and hypoxia not only reduced the mean value of heart rate, but also irregularized cardiac rhythm causing an important increment of heart rate variability (Figure 5b,cii); in comparison, this increase was significantly lower in Cx36KO mice than WT mice especially under conditions of hypercapnia in normoxia (∆VHR = 2.73 ± 0.35 for WT vs. 1.70 ± 0.22 of Cx36KO; *N* = 24 per group, *p* = 0.0163 *t*‐Student test). This high irregularity of heart rhythm in the case of WT mice resulted from the generation of recurrent episodes of transient bradycardia (ETB). During each ETB, heart rate first dropped sharply and then recovered slower during subsequent cardiac cycles (Figure 6ai arrows, and 6bii arrows a–c); the strength of ETBs increased with the time of exposure to high CO_2_ until reaching steady‐state value in a few minutes (Figure 6bi). The cross‐correlation of ETBs onset with ventilation revealed that its generation was phase‐locked with the expiratory phase of breathing cycles (Figure 6biii), probably linked to generation of active expirations (Figure 6ci, E_2_). The number of ETBs generated also increased with the concentration of CO_2_ applied in inhaled air (Figure 7ai, WT), but its frequency of generation did not vary significantly (0.54 ± 0.16 Hz) (Figure 7bi), indicating that the smaller number of ETBs at 4% and 8% than 12% CO_2_ owed to a higher number of failures in its generation. ECG signal‐based Poincaré plots for successive beating intervals (RR_n_ and RR_n+1_) were used to visualize the dynamics of ETBs; each ETB initiated by a discrete shortening of RR interval followed by a larger lengthening of the next interval and a gradual recovery of its basal duration in the successive intervals (Figure 8ai, arrows a–d); the degree of RR lengthening varied from a simple elongation of interval for 4% CO_2_ up to intervals of the double or triple duration for 8% and 12% CO_2_, respectively; this lengthening likely reflects the delay or blockage in the sinoatrial exit of one or two impulses. ETBs in WT mice induced by hypoxic hypercapnia generated at same frequency and were of similar strength than those during normoxic hypercapnia (Figure 7aii, WT; Figure 8bi), but its number decreased up to half after 10 min of exposition to hypoxic hypercapnia (Figure 7aii, WT). In comparison, Cx36KO mice generated significantly fewer ETBs in hypercapnia‐normoxia than WT mice (Figure 6aiii, and Figure 7ai, KO; *N* = 12 per group, *p* < 0.0001 ANOVA) without differences in strength of bradycardic effect (Figure 8aii), frequency of generation (Figure 7biii), and phase‐locking with respiration (Figure 6bvi and Cii). However, the ETBs generated by Cx36KO mice in hypoxic hypercapnia were of much smaller intensity (Figure 6aiv and 6bv, and Figure 8bii) and tended to disappear rapidly with the time of exposition to hypoxic hypercapnia (Figure 7aii, KO).

**FIGURE 9:**
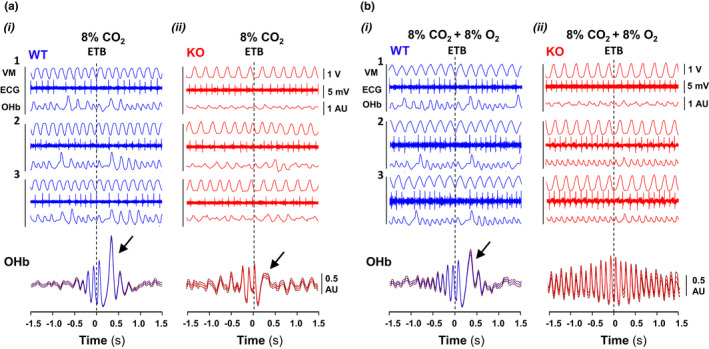
Functional role of ETBs. The impact of ETBs on oxyhemoglobin (OHb) content per cardiac pulse was evaluated in WT and Cx36KO mice (in blue and red) during normoxic and hypoxic hypercapnia (A and B). (i) Raster synchronized with ETB onset of ventilatory motion (Vent), heart beating (ECG), and oxyhemoglobin pulse‐to‐pulse signal (OHb), and (ii) cross‐correlation function of OHb signal with ETBs. ETBs caused a marked transient increase in OHb content per pulse in both phenotypes in hypercapnia‐normoxia (arrows), but during hypoxic hypercapnia only in WT mice

To determine the efficiency of ETBs as a mechanism of cardiorespiratory coordination, we performed the cross‐correlations between ETBs onset and oximetry pulse‐to‐pulse signal; this showed that the oxyhemoglobin content per pulse of WT mice during the cardiac cycles increased markedly in both normoxic and hypoxic hypercapnia (Figure 9ai and 9Bi, arrows), indicating that the physiological function of EBTs is to optimize the gaseous exchange at the alveolar level. The ETBs improved the gaseous exchange because the volume of blood perfusion to the lungs increased due to bradycardia and occurred two ventilations per cardiac cycle (Figure 9ai and 9Bi). In the case of Cx36KO mice, ETBs also enhanced the oxyhemoglobin content per pulse during hypercapnia‐normoxia but not during hypoxic hypercapnia because under this condition the number of EBTs generated decreased significantly, and they caused a much less bradycardic effect (Figure 9aii and 9bii).

### 3.3 Elevated risk of sudden death induced by hypoxic hypercapnia in neonatal Cx36KO mice

**FIGURE 10:**
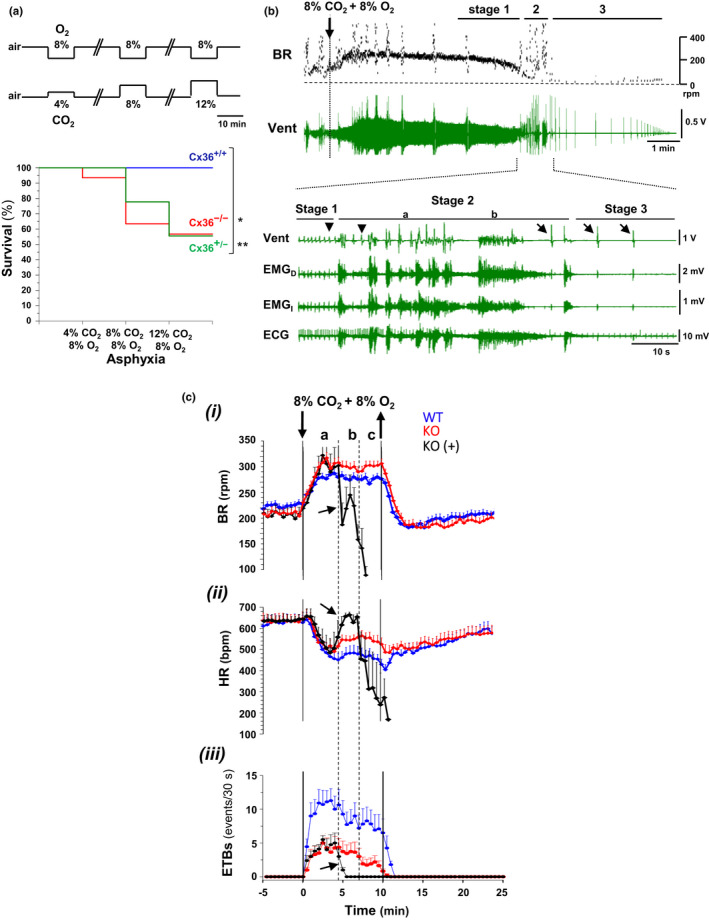
Sudden respiratory arrest induced by hypoxic hypercapnia in the neonatal Cx36‐KO mouse. (A) Survival curves of homozygous (Cx36^−^/Cx36^−^) and heterozygous (Cx36^−^/Cx36^+^) versus wild‐type (Cx36^+^/Cx36^+^) mice submitted to a protocol of severe hypoxia in combination with increasing CO_2_ concentrations (upper); homozygous and heterozygous Cx36KO but not WT mice were susceptible to succumbed by respiratory arrest (0 out 18 Cx36^+^/Cx36^+^, 5 out 9 Cx36^−^/Cx36^+^, and 15 out 26 Cx36^−^/Cx36^−^; *p = 0.0021 Cx36^−^/Cx36^−^ vs. Cx36^+^/Cx36^+^ and **p = 0.0017 Cx36^+^/Cx36^−^ vs. Cx36^+^/Cx36^+^, log‐rank test). (B) Stages leading to sudden respiratory arrest: stage 1, eupneic depression; stage 2, paroxysmal respiratory motor activity; and stage 3, terminal gasping; BR, breathing rate and Vent, respiratory chest motion. Inset, expanded records of respiratory motion (Vent), diaphragm, and intercostal muscles (EMG_D_ and EMG_I_), and cardiac activity (ECG) showing the transition from eupnea to gasping mode (arrowheads and arrows) and the sudden cardiac rate drop occurring during the paroxysmal clonic‐tonic activity (a and b in stage 2). (C) Superimposed average responses to 8% O_2_ and 8% CO_2_ of survival WT and Cx36KO mice (*N* = 20 and *N* = 16 in blue and red), and of Cx36KO mice that succumbed at 7.29 ± 0.51 min (*N* = 8; in black). Early events during eupneic collapse (stage 1, arrows in b) were: the slowdown of breathing rate (BR in i), the acceleration of heart rate (HR in ii), and the suppression of episodes of transient bradycardia (ETBs in iii)

In agreement with the hypothesis of the "triple risk model" of SIDS pathogeny (Filiano & Kinney, 1994), the central defects in the ventilatory control and cardiorespiratory coordination observed in Cx36KO mouse should confer an intrinsic vulnerability for suffering sudden death by asphyxia, as occurs when infant re‐breaths exhaled gases with a high CO_2_ and a poor O_2_ content (Pasquale‐Styles et al., 2007). Neither of the WT mice (18 out 18) died during the serial application of severe hypoxia (8% O_2_) stimuli in combination with increasing CO_2_ concentrations of 4%, 8%, and 12% (Figure 10a); however, the 42% (15 out 26) of homozygous Cx36^−^/Cx36^−^ mice succumbed by respiratory arrest (*p* = 0.0021 for Cx36^−^/Cx36^−^ vs. Cx36^+^/Cx36^+^, log‐rank test). The percentage of sudden death in the heterozygous Cx36^+^/Cx36^−^ mice was similar, the 44% (5 out 9; *p* = 0.0017 Cx36^+^/Cx36^−^ vs. Cx36^+^/Cx36^+^, log‐rank test). A stereotyped cascade of events, in which three principal stages distinguished, led to the respiratory arrest. First, the initially exacerbated eupneic respiration collapsed in frequency and amplitude (Figure 10b stage 1 and C‐i a‐b), and the generation of EBTs stopped, causing the subsequent heart rate acceleration (Figure 10B, ii and iii arrows in b). Then, Cx36KO mice generated a paroxysmal generalized clonic‐tonic motor activity lasting on average 53 + 4 s (*N* = 8) (Figure 10bstage 2); this seizure‐like activity provoked the definitive cessation of eupneic rhythm and the starting of gasping respiration, and the collapse of heart rate (Figure 10b inset). The gasps of very slow frequency (28 + 10 bpm; *N* = 8) and high amplitude restored the automatism of breathing temporally (126 + 49 s; *N* = 8) but they were unable to recover the eupneic respiration and heart rate. Finally, the decrease in amplitude and increase in frequency and duration of gasps marked the endpoint of respiratory failure (Figure 10b/ state 3); the cardiac arrest occurred few minutes later.

## 4 DISCUSSION

Neuronal Cx36 gene deletion resulted during the postnatal period (P14) in serious disturbances in the control of ventilation and cardiorespiratory coordination and therefore, the risk of sudden infant death induced by respiratory stressor increased. With the limitations of knocking out gene technology to elucidate between primary effects and eventual compensatory changes, these results support the idea that electrical connectivity within respiratory networks of the brainstem is essential for the proper functioning of the respiratory center during postnatal peak expression of Cx36 (Belluardo et al., 2000; Solomon, 2003).

### 4.1 Disturbances in the respiratory control and cardiorespiratory coupling

Mice lacking Cx36 show breathing instability at rest and exacerbated chemoreflexes. The rhythmicity of respiratory motor pattern arises from two distinct but interacting generators (Huckstepp et al., 2015, 2016, 2018): an “inspiratory” oscillator located in the pre‐BötC, the kernel of respiration (Smith et al., 1991), that drives the inspiratory rhythm, and a conditional “expiratory” oscillator ubicated in the lateral region of the parafacial respiratory group (pF_L_) that initiates, modulates, and sustains active expiration (Janczewski & Feldman, 2006); breathing at rest is mainly driven by the “inspiratory” oscillator whereas expiration is passive because the “expiratory” oscillator is inhibited (Pagliardini et al., 2011); active expiration recruits under respiratory stressing conditions triggered fundamentally by hypercapnia (Abdala et al., 2009; Leirão et al., 2018). Breathing instability of Cx36KO mice at rest varied from a slower and more irregular respiratory rhythm to a periodic Cheyne–Stokes respiration with recurrent periods of hypopnea and hyperpnea; this less robustness of the inspiratory rhythm may be accounted for by the loss of electrical coupling and subsequent desynchronization of the rhythmogenic neurons of preBötC (Rekling et al., 2000). According to the “loop gain” theory of a ventilatory system with negative feedback control (Khoo et al., 1982), the periodic Cheyne–Stokes respiration also require an abnormally increased sensitivity to CO_2_ at apnea level causing the repeating overshoot/undershoot of breathing drive. This increased CO_2_ sensitivity is precisely an outstanding feature of the Cx36KO mouse responses to hypercapnic stimuli; under these conditions, the hypersensitivity to CO_2_ resulted in a stronger inspiratory–expiratory coupling in Cx36KO than WT mice, generating faster breathing cycles and more intensive phasic activations in an anti‐phase coupling of inspiratory and expiratory musculatures. Although Cx36 expresses in the carotid bodies (Frinchi et al., 2013), the similar respiratory depression caused by hyperoxia in both genotypes suggests a negligible contribution of peripheral chemoreception afferents to the hypersensitivity to CO_2_ of Cx36KO mice, indicating a central origin. Because Cx36 gap junctional channels and hemichannels are not CO_2_ sensitive (González‐Nieto et al., 2008; Huckstepp et al., 2010), the Cx36 suppression should not change the sensitivity to CO_2_. However, an upregulation of the CO_2_‐sensitive Cx26, Cx32, and Cx30 (Huckstepp et al., 2010) cannot rule out; in a previous study was reported that Cx30.2, Cx37, Cx43, Cx45, pannexin 1, and pannexin 2 levels remain unchanged in the Cx36KO mouse (Jacobson et al., 2010). Given the preferential expression of Cx36 in inhibitory neurons (Belluardo et al., 2000; Deans et al., 2001; Nagy et al., 2018), it is reasonable to propose that the increase in CO_2_ sensitivity may be accounted by a mechanism of disinhibition, similar to that underlying the impairment of gamma rhythm in Cx36KO mice based on that in absence of Cx36 synapses the neuronal activity of uncoupled inhibitory networks desynchronizes and therefore, the effectiveness of inhibitory network output decreases (Hormuzdi et al., 2001). In this line, it has been reported that hypercapnia activates by a mechanism of disinhibition to the conditional “expiratory” oscillator of pF_L_ to evoke active expiration (Britto & Moraes, 2017). The extensive direct inhibitory projections from BötC, preBötC, and rostral ventral respiratory group to pF_L_ (Biancardi et al., 2021), which likely express Cx36 (Parenti et al., 2000; Solomon, 2003), can be the source of this mechanism of disinhibition.

The deficiency of Cx36 also disturbed the autonomic regulation of the cardiovascular system during chemoreflexes. In this regard, an outstanding finding of this study is the identification of a new mechanism of cardiorespiratory coupling induced by hypercapnia consistent in the generation of recurrent episodes of transient bradycardia (ETBs) for improving arterial O_2_ saturation during asphyxia. The sine‐wave‐like undulating pattern of the heart rate observed in antepartum, intrapartum, and neonatal monitoring (Nageotte, 2015) would be the equivalent in humans of ETBs identified now in rodents. The abrupt and intense drop in heart rate during ETBs must account by an intensive discharge from the cardiac vagal preganglionic neurons (CVN) of the nucleus ambiguous that controls the heart rate (Gourine et al., 2016), of sufficient intensity to block the sinus node exit of one or two atrial impulses. Our data indicate that respiration triggers this tetanic discharge of CVNs since the onset of ETBs is phase‐locked with the expiratory phase of the breathing cycle, suggesting a causal link between the mechanism of generation of active expirations and ETBs. At resting conditions, the acceleration and deceleration of heart rate during inspiration and expiration that underlying respiratory sinus arrhythmia (RSA) results respectively of inhibition of cardiac vagal preganglionic neurons from preBötC during the second part of expiration and the first part of inspiration (Menuet et al., 2020) and excitation from the pontine Kölliker‐Fuse nucleus during post‐inspiration (Farmer et al., 2016). It is obvious that during the CO_2_‐induced ETBs this pattern of respiratory‐related inputs to CVNs should suffer a drastic reconfiguration that deserves further analysis; in comparison to RSA modulation within each respiratory cycle, ETBs provoke a ≈100 fold stronger modulation of heart rate lasting 6–9 respiratory cycles or multiples of these values, depending on whether the ETBs generates at the fundamental frequency (≈0.5 Hz) or its harmonics. The combination of hypoxia with hypercapnia exerts an antagonistic effect on the CO_2_‐induced ETBs but, while hypoxic hypercapnia in WT mice only reduces the number of ETBs without affecting the intensity of the bradycardic effect, hypoxia in mice lacking Cx36 reduces substantially both, the bradycardia effect and the generation of ETBs, causing a more pronounced desaturation of arterial O_2_.

### 4.2 Neonatal Cx36KO mouse as a model of SIDS

The increased risk of sudden death in the neonatal Cx36KO mouse is consistent with the generally accepted hypothesis that an important subset of SIDS is caused by disturbances in the central control of ventilation and autonomic regulation of cardiorespiratory coordination (Harper & Kinney, 2010). Regarding the nature of the respiratory stressor causing the SIDS, our experiments indicate that the combination of low O_2_ with high CO_2_ results more deleterious than hypoxia or hypercapnia separately since by themselves they did not induce sudden death; this is likely because the disturbances in the generation of ETBs worsen arterial O_2_ saturation; thus, a defective generation of ETBs, like are those previously reported defects in sighs, arousals, and gasping (Garcia et al., 2013), would be considered as a physiological biomarker of susceptibility in SIDS. Abnormalities of breathing control may cause SIDS either by hyporesponsive chemoreflexes that may allow hypoxia to persist without an adequate response or by destabilizing hyperresponsive chemoreflexes (Cummings et al., 2011; Gauda et al., 2007; Rossor et al., 2018) as seem to be the case of the Cx36KO mice. In the stereotyped cascade of events leading to the respiratory arrest in the neonatal Cx36KO mice, we have identified two pathophysiological determinants not previously reported: the early collapse in the generation of EBTs, which accelerates heart rate and worsens the O_2_ saturation, and the generation of a paroxysmal clonic‐tonic generalized motor discharge that provokes the definitive cessation of eupneic rhythm and the starting of gasping respiration, and precipitates the collapse of heart rate. In light of this finding, we postulate that fatal respiratory arrest in SIDS could be mediated by a hypoxia‐induced spreading‐like depression in the brainstem (Funke et al., 2009), a self‐propagating depolarizing wave that slowly depolarizes neurons and ultimately silences neural networks. In this regard, the Cx36KO mouse model would be suitable to investigate the putative role of brainstem spreading depolarization in the SIDS pathophysiology. Gasping is considered as the last resource of “self‐resuscitation” to restore heart rate and eupneic respiration (Garcia et al., 2013); however, like in SIDS victims (Sridhar et al., 2003), gasping in Cx36KO mice was not effective as a mechanism of autoresuscitation.

## CONFLICT OF INTEREST

LFP, AMC, JMI, JAB, CM, DP, DLP, and LCB declare no financial or personal competing interest in connection with the manuscript.

## AUTHOR CONTRIBUTIONS

Luis C. Barrio, Leonel F. Pérez‐Atencio, and David Pestaña conceptualized the study and designed the experiments. Leonel F. Pérez‐Atencio, Ana M. Casarrubios, Cristina Medrano, Juan A. Barios, and Luis C. Barrio collected, assembled, analyzed, and interpreted the data. David L. Paul and José M. Ibarz contributed respectively in animals and home‐made devices. Luis C. Barrio wrote the manuscript and all authors approved its final version.
